# Nitro-fatty acids as activators of hSIRT6 deacetylase activity

**DOI:** 10.1074/jbc.RA120.014883

**Published:** 2021-01-13

**Authors:** Mara Carreño, Mariana Bresque, Matías R. Machado, Leonardo Santos, Rosario Durán, Darío A. Vitturi, Carlos Escande, Ana Denicola

**Affiliations:** 1 Laboratorio de Fisicoquímica Biológica, Instituto de Química Biológica, Facultad de Ciencias, and Centro de Investigaciones Biomédicas (CEINBIO), Universidad de la República, Montevideo, Uruguay; 2 Laboratorio de Patologías del Metabolismo y el Envejecimiento, Institut Pasteur de Montevideo, Montevideo, Uruguay; 3 Laboratorio de Simulaciones Biomoleculares, Institut Pasteur de Montevideo, Montevideo, Uruguay; 4 Unidad de Bioquímica y Proteómica Analíticas, Institut Pasteur de Montevideo, Instituto de Investigaciones Biológicas Clemente Estable, Montevideo, Uruguay; 5 Department of Pharmacology and Chemical Biology; Heart, Lung, Blood and Vascular Medicine Institute, and Center for Critical Care Nephrology, University of Pittsburgh School of Medicine, Pittsburgh, Pennsylvania, USA

**Keywords:** activator, sirtuin, SIRT6, fatty acids, nitro-fatty acids, docking, histone deacetylation, cysteine covalent modification, Michael addition, enzyme activation, histone deacetylase (HDAC), enzyme mechanism, enzyme structure, enzyme kinetics, fatty acid, cysteine covalent-modification, enzyme activator, nitro-fatty acid

## Abstract

Sirtuin 6, SIRT6, is critical for both glucose and lipid homeostasis and is involved in maintaining genomic stability under conditions of oxidative DNA damage such as those observed in age-related diseases. There is an intense search for modulators of SIRT6 activity, however, not many specific activators have been reported. Long acyl-chain fatty acids have been shown to increase the weak *in vitro* deacetylase activity of SIRT6 but this effect is modest at best. Herein we report that electrophilic nitro-fatty acids (nitro-oleic acid and nitro-conjugated linoleic acid) potently activate SIRT6. Binding of the nitro-fatty acid to the hydrophobic crevice of the SIRT6 active site exerted a moderate activation (2-fold at 20 μm), similar to that previously reported for non-nitrated fatty acids. However, covalent Michael adduct formation with Cys-18, a residue present at the N terminus of SIRT6 but absent from other isoforms, induced a conformational change that resulted in a much stronger activation (40-fold at 20 μm). Molecular modeling of the resulting Michael adduct suggested stabilization of the co-substrate and acyl-binding loops as a possible additional mechanism of SIRT6 activation by the nitro-fatty acid. Importantly, treatment of cells with nitro-oleic acid promoted H3K9 deacetylation, whereas oleic acid had no effect. Altogether, our results show that nitrated fatty acids can be considered a valuable tool for specific SIRT6 activation, and that SIRT6 should be considered as a molecular target for *in vivo* actions of these anti-inflammatory nitro-lipids.

Sirtuins are a family of NAD^+^-dependent lysine deacylases critical for maintaining cellular and organ homeostasis (1, 2, 3, 4). This family includes seven sirtuins (SIRT1–7) with different subcellular location, enzymatic activities, substrate proteins, and biological functions, as well as different key roles in metabolism, nutrition, aging, inflammation, and cancer (5, 6, 7, 8). SIRT6 is primarily located in the nucleus along with SIRT1 and SIRT7, whereas SIRT2 is cytosolic, and SIRT3, SIRT4, and SIRT5 are found in the mitochondria (9).

SIRT6 is implicated in the regulation of diverse physiological processes such as maintenance of genomic stability, inflammation, and energy metabolism (10, 11, 12). Overexpression of SIRT6 increased lifespan in male mice, preserved glucose tolerance, and attenuated adipose tissue inflammation (13, 14, 15). More recently, overexpression of SIRT6 showed neuroprotection in an animal model of spinal cord injury (16). Conversely, mice globally deficient in SIRT6 are hypoglycemic, suffer lymphopenia, premature aging, and die within 1 month (17). Tissue-specific SIRT6 ablation in adipocytes, macrophages, or pancreatic beta-cells, sensitizes mice toward high-fat diet-induced obesity and insulin resistance (18, 19, 20). Specific neural SIRT6-deleted mice did not die from hypoglycemia, but displayed postnatal growth retardation and ultimately became obese (21). In these latter mice, hyperacetylation of histone H3 lysine 9 (H3K9) and lysine 56 (H3K56) were observed in various regions of the brain. In fact, H3K9 and H3K56 are the main molecular targets for SIRT6 activity *in vivo* (22, 23, 24) with recent reports also including H3K18 (25).

In addition to deacetylating histone H3, SIRT6 can also bind and regulate nonhistone proteins such as the transcription factor HIF-1α (26), catalyze intramolecular mono-ADP-ribosylation (27), and mediate deacylation of long-chain fatty acyl groups (*e.g.* myristoylated tumor necrosis factor-α (28)). As recently reviewed by Klein and Denu (29) the physiological substrates, biological activities, and specific cellular functions of SIRT6 still need further investigation.

All sirtuins share a highly conserved globular catalytic core of 275 amino acids but the N and C termini are highly disordered and differ significantly among family members (30, 31, 32). The active site comprises three structural pockets A, B, and C, with the latter having the most hydrophobic character and the largest number of highly conserved residues. Two domains flank the active site, a large Rossmann-fold where co-substrate NAD^+^ binds, and a smaller Zn^2+^-binding motif (Fig. 1) (33). In particular, SIRT6 displays a more open structure in which the two domains are more distant than in other isoforms (34). Accommodation of a long-chain acyl group in the C-pocket favors the domains to close up, thus resulting in better deacylase than deacetylase activity (28, 35). The N- and C-terminal regions in SIRT6 are short, less structured and flexible. The C terminus is required for nuclear localization, whereas the N terminus, rich in positive residues, is important for both chromatin association and for intrinsic catalytic activity because its deletion severely compromised H3K9 and H3K56 deacetylation (27, 36, 37). The mechanism of catalysis includes the binding of cofactor NAD^+^ and the acylpeptide in the right orientation so that the amide of the acylated lysine can attack the anomeric carbon of the ribose via an SN2-type mechanism, releasing nicotinamide and forming an alkylamidate intermediate (35, 38, 39). A conserved histidine residue in the active site functions as a general base to catalyze the release of the acylated-ADPR and lysine peptide products (40, 41). Thus, in sirtuins, catalysis depends on this histidine residue (His-133 in the human SIRT6 isoform, hSIRT6) and not on Zn^2+^ as in other histone deacetylases (HDAC class I, II, and IV). In this regard, the zinc atom has a purely structural role and it is coordinated by four cysteine residues in a C*XX*C motif. In the case of hSIRT6 these are Cys-141, Cys-144, and Cys-166, Cys-177, where the insertion of 10 residues (167 to 176) forming a flexible loop, is unique for SIRT6 (Fig. 1).

**Figure 1:**
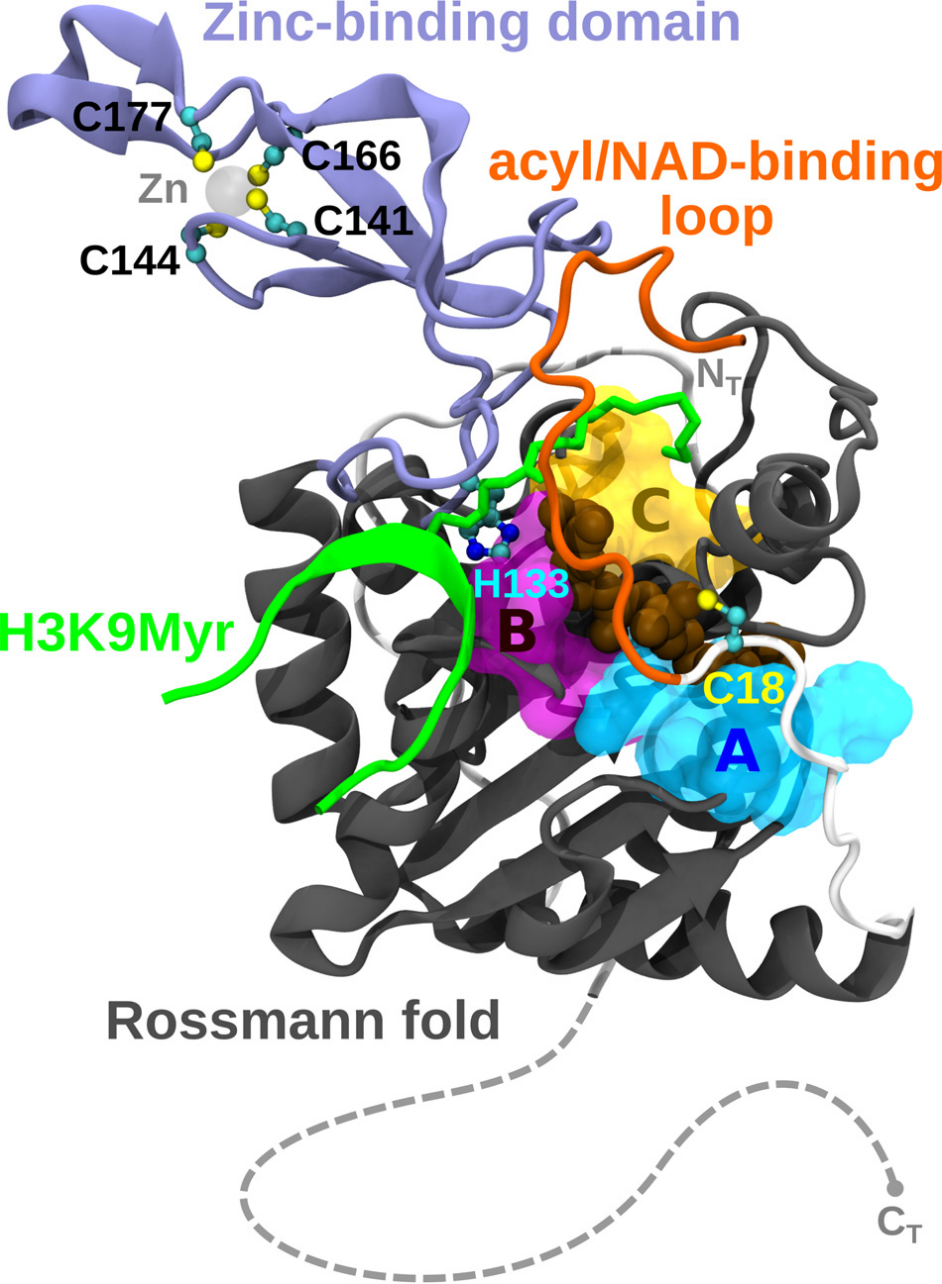
**Human SIRT6 structure.** Molecular representation of hSIRT6 in complex with a myristoylated H3 peptide at Lys-9 (H3K9Myr) and a co-substrate (PDB code 3ZG6). Structural features such as functional domains, A, B, C pockets and relevant residues are indicated. The location of N and C termini (N_T_ and C_T_, respectively) is also shown. Continuation of the peptide chain structure toward C_T_ is sketched as a guide for helping visualization.

Given its importance in the maintenance of energy metabolism and DNA stability, the discovery of novel pharmacological agents that boost SIRT6 activity can have great potential for treatment of diabetes, arthritis, cardiovascular, and neurodegenerative diseases (42). As a result, there is an intense search for modulators of SIRT6 activity, however, not many specific activators have been reported. Interestingly, Feldman *et al.* (43) demonstrated that the low *in vitro* deacetylase activity of SIRT6 could be activated in the presence of free long-chain fatty acids, particularly oleic and linoleic acid (5-fold increase at 100 μm), and that this activation was not displayed by SIRT1. Similarly, polyphenols were shown to either inhibit (catechins) or weakly activate (anthocyanidins, 2-fold increase at 100 μm) SIRT6 activity but these responses are similarly weak (44, 45). In addition, two synthetic small molecule activators have been identified, a pyrrole[1,2-*a*]quinoxaline derivative (UBCS039) that achieved a 2-fold increase in activity at 100 μm (46) and MDL-800, a more effective pharmacological activator (22-fold at 100 μm) (47), both binding at a distal region of the fatty acyl substrate. Finally, a targeted screen of fatty acids and lipid-like molecules identified other activators with >15-fold activation at 100 μm such as oleoyl-lysophosphatidic acid (48-fold) and 2-(3-chloro-4-(2,4-dichlorobenzamido)phenyl)-1,3-dioxoisoindoline-5-carboxylic acid (CL-4, 18-fold) (48).

Nitro-fatty acids (NO_2_-FA) are detected in diverse species, including humans (49, 50, 51) and have been extensively shown to exert cellular protective actions and to protect against metabolic disorders and inflammation (52). NO_2_-FA are both derived from the diet and can also be formed endogenously by the olefinic nitration of unsaturated fatty acids thus reaching nanomolar concentrations in the plasma of healthy subjects (50, 53). In addition, dietary supplementation with nitrite and CLA (conjugated linoleic acid, also known as rumenic acid) have been shown to increase the levels of NO_2_-CLA in plasma, urine, and tissues (49, 50). Importantly, the nitration of unsaturated fatty acids results in the formation of an electrophilic nitroalkene moiety that can react via Michael addition with nucleophilic residues, thus resulting in post-translational modification (PTM) of transcription factors and enzymes (52, 54, 55, 56). These post-translational modifications alter protein structure and function, resulting in signaling actions that include Nrf2-regulated gene induction, inhibition of NF-κB-dependent proinflammatory cascades, and modulation of enzyme activities such as xanthine oxidoreductase inhibition and pro-matrix metalloproteinases-7 and -9 activation (57, 58, 59, 60, 61). Considering the presence of a nucleophilic histidine in the active site of sirtuins, as well as of several cysteine residues with known structural roles (zinc-binding motif), we explored the potential of NO_2_-FA to exert structural and functional changes in these proteins. Herein we report that the nitrated fatty acids nitro-oleic and nitro-conjugated linoleic acid (Fig. 2*A*) potently activate SIRT6 deacetylase activity via covalent modification of the isoform-specific Cys-18 in the N-terminal domain.

**Figure 2:**
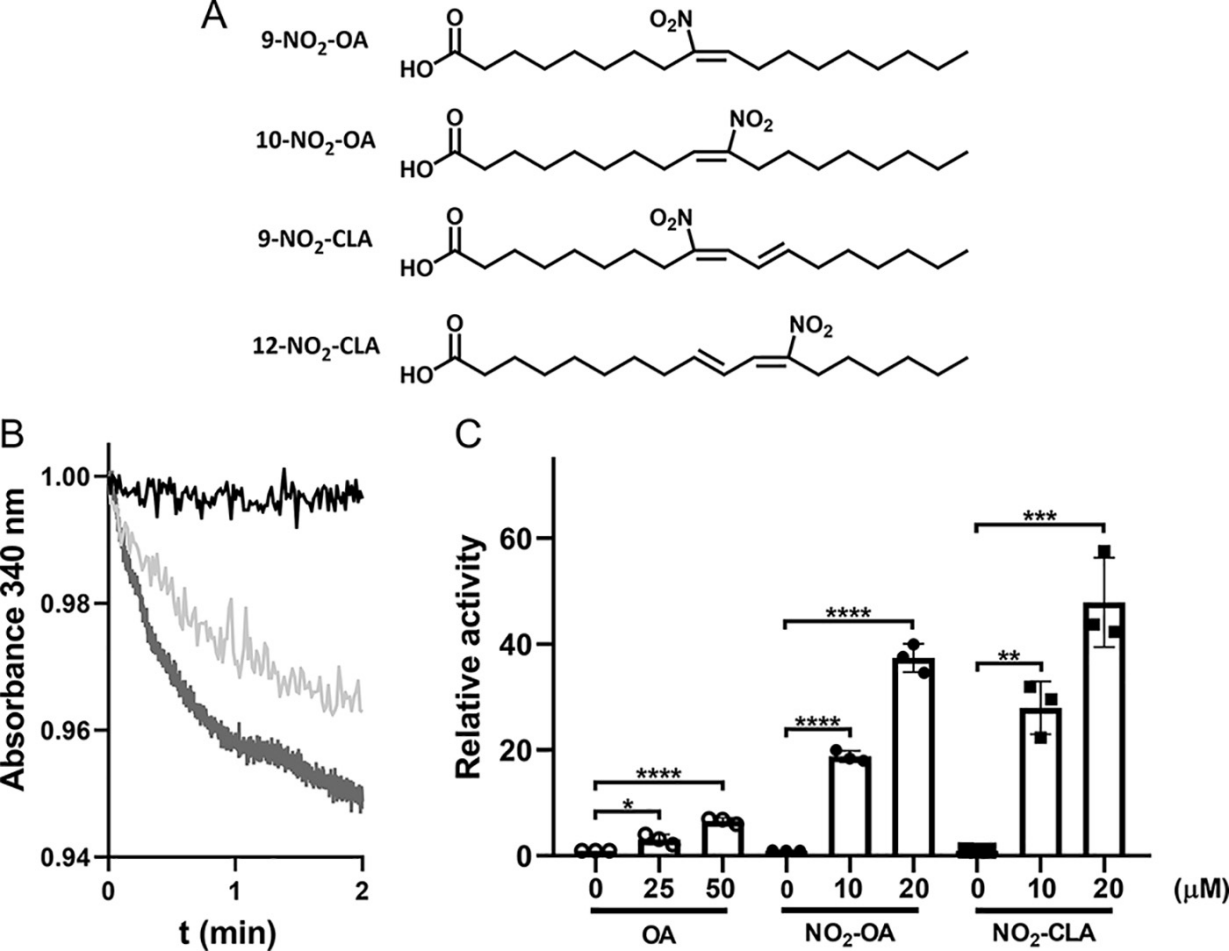
**Effect of fatty acids and nitro-fatty acids on *in vitro* deacetylase activity of hSIRT6.***A,* chemical structures of 9- and 10-nitro-oleic acids (NO_2_-OA), 9- and 12-nitro-conjugated linoleic acids (NO_2_-CLA). Experiments were performed using the racemic mixtures 9- and 10-NO_2_-OA, and 9- and 12-NO_2_-CLA in the presence of 1 μm SIRT6. *B,* representative runs of deacetylase activity measured using the coupled assay for untreated hSIRT6 (*black*), hSIRT6 incubated with oleic acid (50 μm, *light gray*), and hSIRT6 incubated with nitro-oleic acid (20 μm, *dark gray*). *C,* fold-change in SIRT6 deacetylase activity after incubation with the indicated concentrations of OA (○), NO_2_-OA (●), and NO_2_-CLA (■). *Error bars* represent S.D. of at least three replicates. *, *p* < 0.1; **, *p* < 0.01; ***, *p* < 0.001; ****, *p* < 0.0001 ANOVA test.

## Results

### Nitro-fatty acids stimulate the deacetylase activity of hSIRT6 in vitro

The *in vitro* deacetylase activity of hSIRT6 is low under basal conditions (*k*_cat_ = 0.003 s^−1^, *K_m_* = 400 μm) and the activation by oleic acid (OA) was confirmed as previously reported (Fig. 2*B*). Notably, incubation with a racemic mixture of nitro-oleic acid (NO_2_-OA) resulted in potent activation of the deacetylase activity, with a fold of activation significantly higher than that achieved by the corresponding non-nitrated fatty acid (Fig. 2*C*). In this regard, preincubation of SIRT6 with a 20-fold excess of OA increased deacetylase activity (H3K9Ac) by 2-fold, whereas the same treatment with NO_2_-OA resulted in 40-fold activation. Similar results were obtained with nitro-conjugated linoleic acid (NO_2_-CLA), the most abundant endogenous NO_2_-FA in humans (Fig. 2*C*). No activation was observed for the SIRT6 deacylase (H3K9Myr) activity (*k*_cat_ = 0.008 s^−1^, *K_m_* = 20 μm). Similarly, no effect was observed by either OA (43) or up to 100 μm NO_2_-OA on the deacetylase and demyristoylase activities of SIRT1 (data not shown).

**Figure 3:**
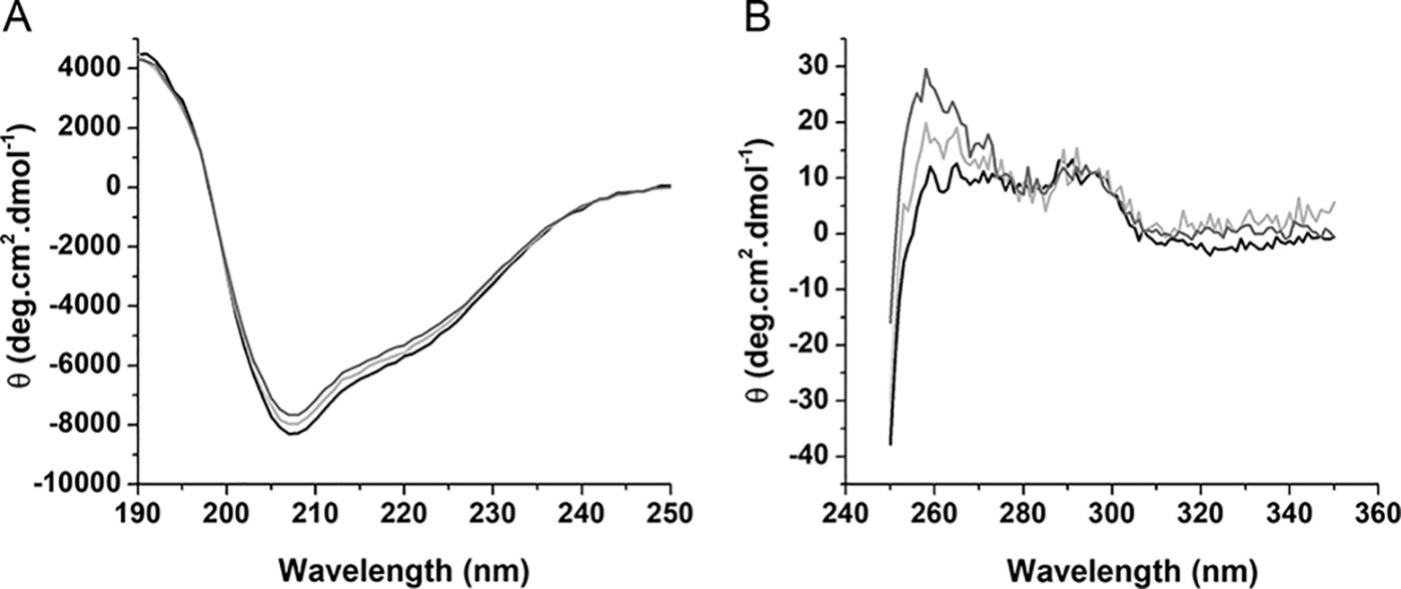
**CD of hSIRT6.** Far-UV (*A*) and near-UV (*B*) CD spectra of hSIRT6 (3 and 10 μm, respectively) after 30 min incubation at 37 °C in the absence (*black*) or presence of either 5-fold excess of OA (*light gray*) or NO_2_-OA (*dark gray*). Spectra were normalized for comparison as detailed under “Experimental procedures.”

Incubation with either OA or NO_2_-OA did not significantly alter the secondary structure of hSIRT6, but a conformational change was evidenced by near-UV CD. More specifically, a change in the asymmetric environment of the aromatic residues and disulfides was observed, in particular, an ellipticity increase around 260 nm (Fig. 3).

### Reaction of nitro-fatty acids with hSIRT6 cysteine residues

**Figure 4:**
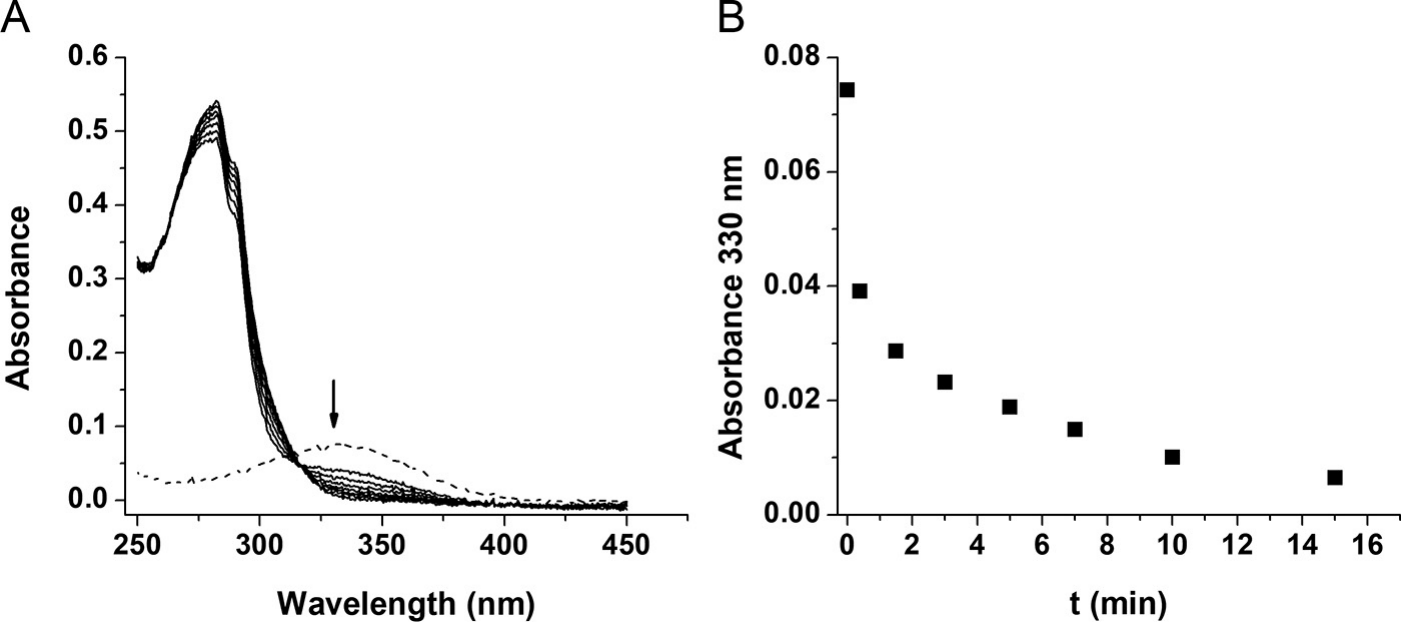
**Consumption of NO_2_-CLA by hSIRT6.***A,* UV-visible spectra of hSIRT6 (*solid black line*) with a characteristic peak at 280 nm, NO_2_-CLA alone (*dashed line*) with a characteristic peak at 330 nm, and of a mixture of 15 μm hSIRT6 with 10 μm NO_2_-CLA. *B,* change in absorbance at 330 nm with time after mixing hSIRT6 with NO_2_-CLA (1.5:1).

**Figure 5:**
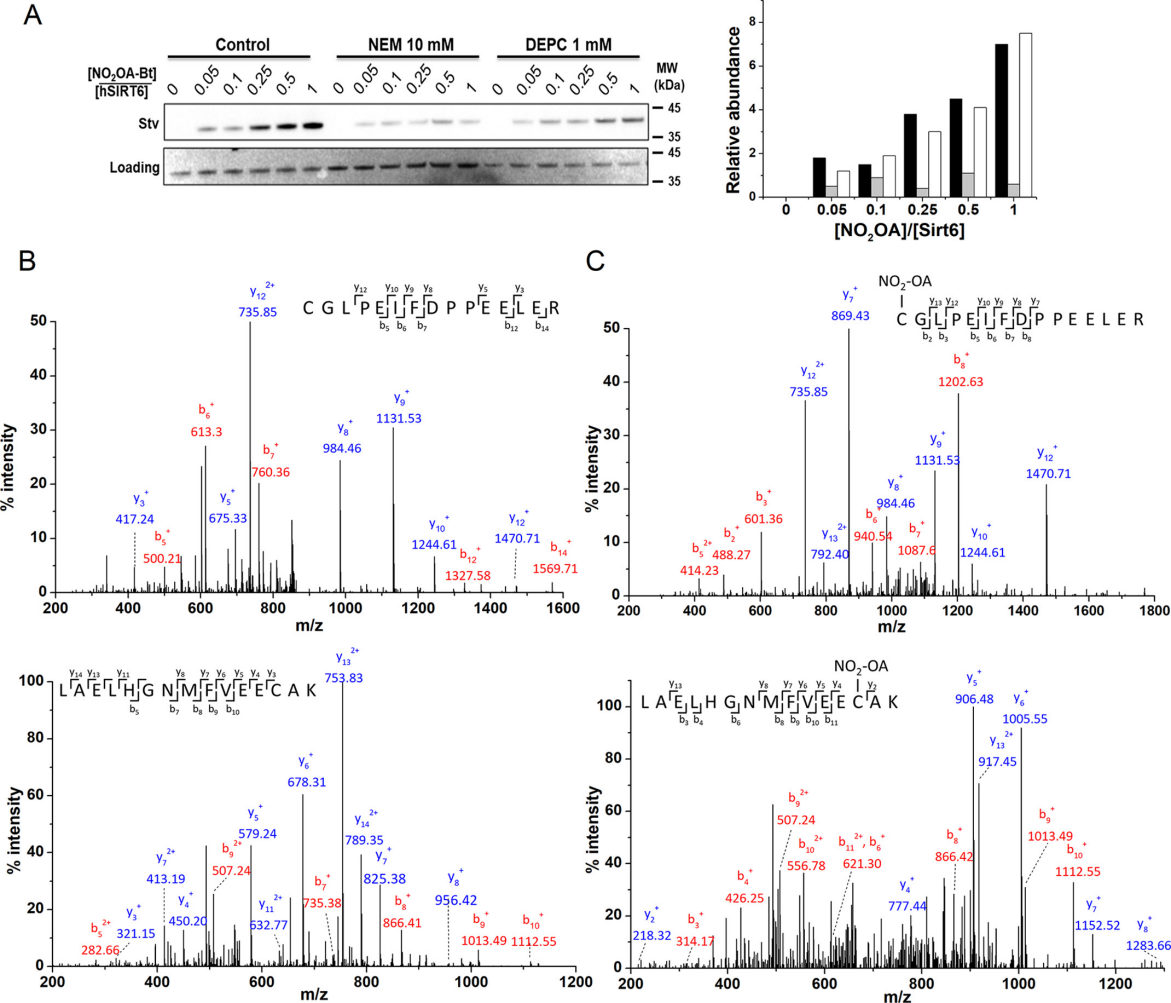
**Identification of hSIRT6 residues modified by NO_2_-OA.***A,* untreated hSIRT6 (*control*), and hSIRT6 treated with either NEM or DEPC to block cysteine and histidine residues, respectively, was incubated with the indicated excess of biotinylated-NO_2_-OA and adduct formation detected using streptavidin-HRP (*upper panel*). Ponceau S staining was used as a loading control (*lower panel*) for densitometric analysis: control (*black*), NEM-treated SIRT6 (*gray*), and DEPC-treated SIRT6 (*white*). One of two consistent experiments is shown. *B,* MS spectra of untreated-hSIRT6 peptides obtained from tryptic digestion (2 h at 37 °C). The *upper panel* corresponds to the fragmentation of the doubly charged peptide sequence containing Cys-18 (CGLPEIFDPPEELER; monoisotopic *m/*z: 872.73) and the *lower panel* corresponds to triply charged sequence containing Cys-141 (LAELHGNMFVEECAK; monoisotopic *m/z:* 564.34). *C,* MS/MS spectra of NO_2_-OA–treated SIRT6 (×5) ions with Δm = 327.24. *Upper panel* corresponds to the doubly charged peptide sequence containing Cys-18 (CGLPEIFDPPEELER; monoisotopic *m/z:* 1036.21) and *lower panel* corresponds to the triply charged sequence containing Cys-141 (LAELHGNMFVEECAK; monoisotopic *m/z*: 673.56). The spectra are representative of three independent experiments. *y-* and *b-*ions detected by MS/MS analysis for each tryptic peptide.

Electrophilic NO_2_-FA, such as NO_2_-OA and NO_2_-CLA, can react with nucleophilic protein residues via reversible Michael addition (54, 56). Because hSIRT6 has a catalytic histidine residue, His-133, the incubation with NO_2_-FA could result in inactivation. Taking advantage of the change in absorption of NO_2_-CLA with environment polarity (λ_max_ = 330 nm in water *versus* λ_max_ = 312 nm in methanol), the binding of this nitro-lipid to hSIRT6 was investigated. As shown in Fig. 4, the blue shift expected in the UV-visible spectrum for NO_2_-CLA binding to a hydrophobic site in the enzyme was not observed. Instead, a time-dependent decrease in absorption at 330 nm was detected, suggesting a reaction between NO_2_-CLA and a nucleophilic protein residue. To explore which nucleophilic residues were involved in this reaction, hSIRT6 was treated with NEM or DEPC to block cysteines or histidines, respectively, followed by incubation with biotinylated-NO_2_-OA (Bt-NO_2_-OA). Streptavidin-HRP adduct blotting strongly suggested the occurrence of a covalent interaction between NO_2_-OA and cysteine residues in hSIRT6 (Fig. 5*A*). ESI ionization MS/MS analysis further confirmed this observation and identified Cys-141 (one of the cysteines coordinating Zn^2+^) and Cys-18 at the N-terminal domain, as the reactive targets (Fig. 5, *B* and *C*). Interestingly, despite the confirmation of adduct formation between NO_2_-OA and Cys-141, as well as the conformational change evidenced by CD spectra (Fig. 3), no loss of zinc was detected from hSIRT6 upon incubation with either NO_2_-FA (data not shown).

### N-terminal Cys-18 is responsible for hSIRT6 nitro-fatty acid activation

**Figure 6:**
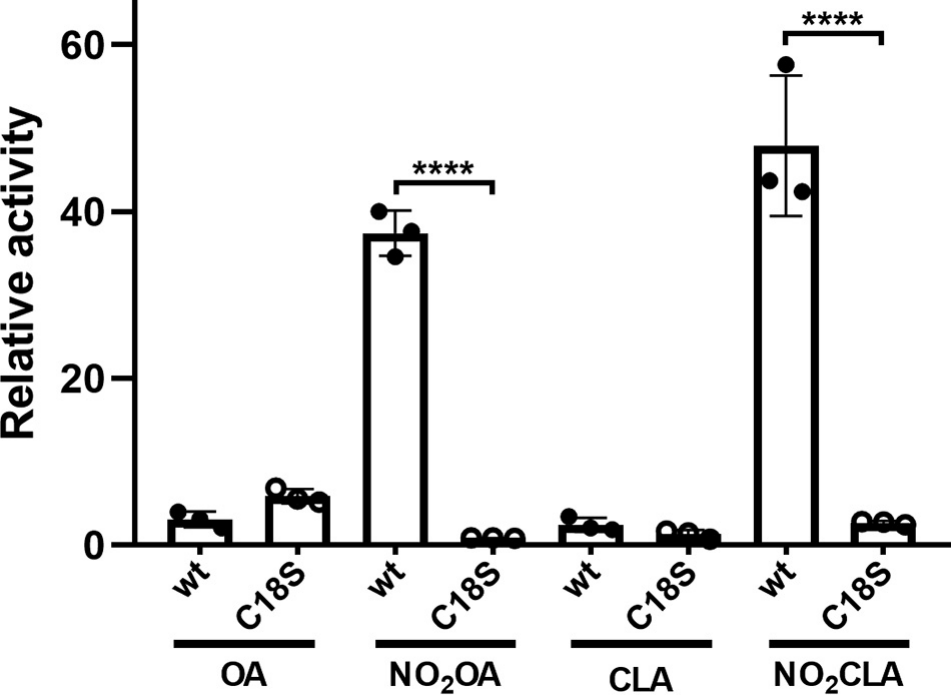
**Effect of fatty acids and nitro-fatty acids on *in vitro* deacetylase activity of WT and C18S hSIRT6.** Fold-change in SIRT6 deacetylase activity after incubation of 1 μm WT hSIRT6 (●) or C18S mutant (○) with 20 μm OA, NO_2_-OA, CLA, or NO_2_-CLA. *Error bars* represent S.D. of three replicates. ****, *p* < 0.0001 ANOVA test.

**Figure 7:**
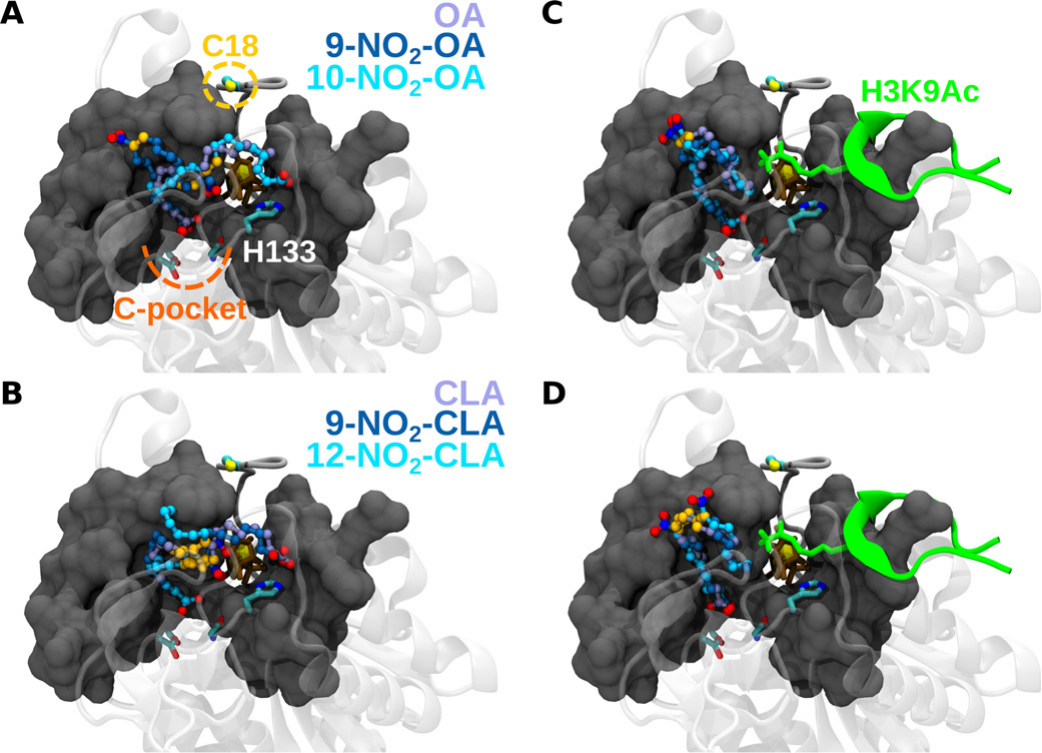
***In silico* docking experiments.***A,* best poses after docking OA and NO_2_-OA into the active site of hSIRT6. The carbon backbone is colored according to the docked molecule. Oxygen and nitrogen atoms are *red* and *blue*, respectively, whereas unsaturations are shown in *gold*. The co-substrate is represented with *brown sticks*. Relevant residues in the protein are pointed out. The molecular surface of the active site is shown in *gray*, whereas the reminding structural context of the protein is shown as a *transparent* cartoon. *B,* same as *panel A* for CLA and NO_2_-CLA. *C*, best poses after docking OA and NO_2_-OA considering the presence of an acetylated H3 peptide at Lys-9 (H3K9Ac). *D,* same as *panel C* for CLA and NO_2_-CLA.

**Figure 8:**
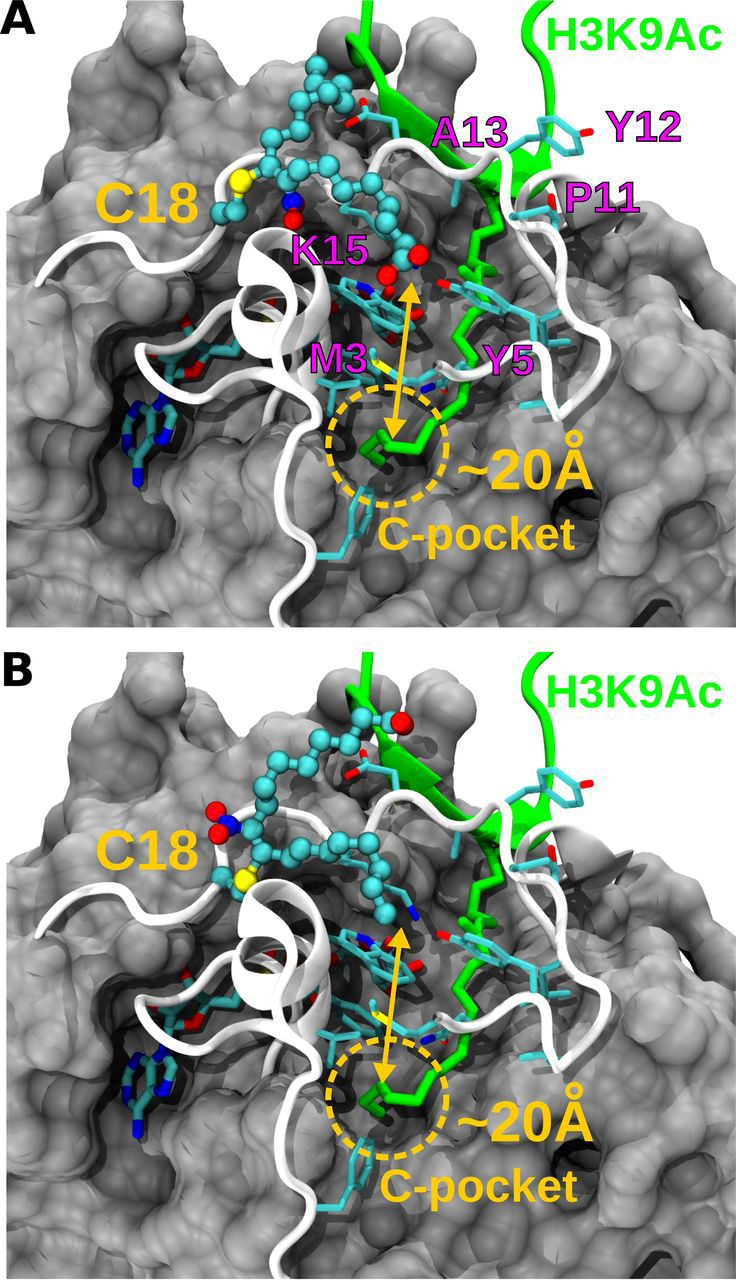
**Molecular modeling of nitro-fatty acid conjugation to Cys-18.***A,* molecular representation of one possible conformation of the Michael adduct between Cys-18 and 9-NO_2_-OA. The nitro-fatty acid is shown in *balls and stick*, whereas nearby protein residues are drawn in *sticks*. Residues that may form an exposed hydrophobic patch at the surface of the acyl/co-substrate–binding loop are labeled in *magenta*. The location and distance to the C-pocket is indicated. *B,* same as *panel A* but showing other alternative conformation that the same adduct may adopt.

Because MS analysis revealed Michael adduct formation with cysteine residues and in particular the N-terminal Cys-18, the effect of NO_2_-FA was assayed on hSIRT6 mutant C18S. Interestingly, the level of activation achieved on the mutant was similar to the effect of the corresponding non-nitrated fatty acid (2-fold). This result suggested that both nitrated and non-nitrated fatty acids bind to the same pocket in the mutant. However, the significant 40-fold activation observed with the WT protein was lost in the absence of adduct formation with Cys-18 (Fig. 6). Molecular docking of NO_2_-OA or NO_2_-CLA into the active site of hSIRT6 in the absence of a H3K9Ac peptide showed that the electrophilic nitroalkene moiety is distant from the catalytic histidine (Fig. 7, *A* and *B*), thus providing an explanation for the non-inhibitory activity of NO_2_-FA on hSIRT6 activity. Furthermore, docking poses of NO_2_-OA or NO_2_-CLA into the active site in the presence of H3K9Ac peptide were similar to those observed with OA and CLA, consistent with ∼2-fold activation observed with of the C18S mutant (Fig. 7, *C* and *D*). The solvent-exposed conformation of CLA's chromophore group in the resulting poses may agree with observations made in its absorption spectrum (Fig. 4). In all cases, Cys-18 was always found far from the nitroalkene group, thus preventing a possible adduct formation with the docked NO_2_-FA in the active site of the WT protein. Molecular modeling of a Cys-18 adduct with 9-NO_2_-OA showed that neither the carboxylic nor the acylic ends of the NO_2_-FA were able to reach the C-pocket of hSIRT6 (∼20 Å away), on the contrary, they should remain close to the acyl/co-substrate–binding loop (Fig. 8).

### Binding of hSIRT6 to nitro-fatty acid at the cellular level

**Figure 9:**
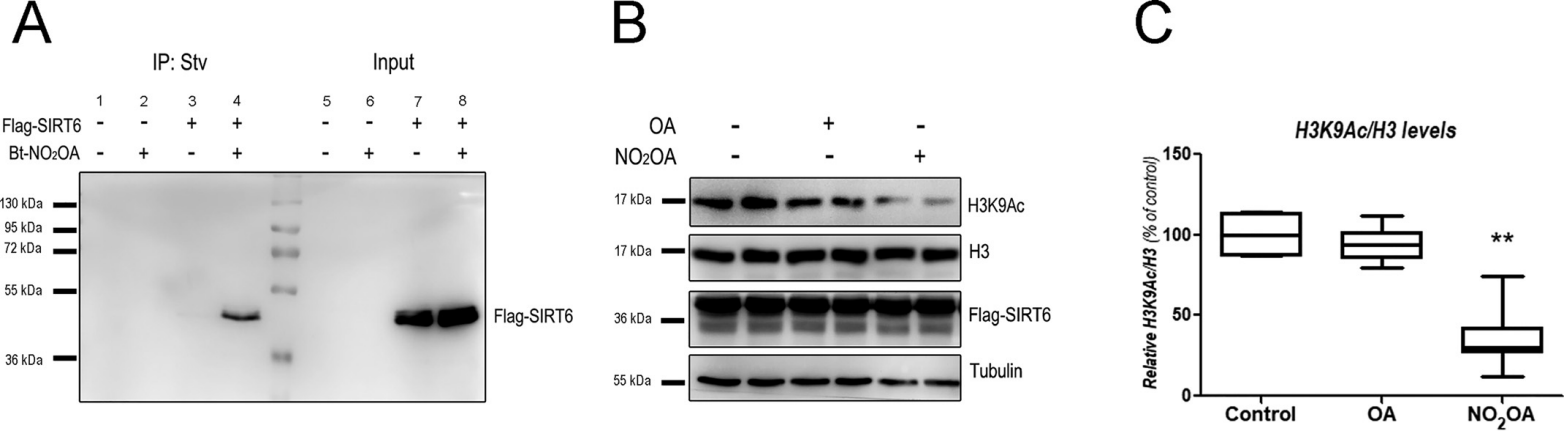
***In situ* covalent modification of hSIRT6 by NO_2_-OA promotes H3K9 deacetylation in cells.***A,* HEK293T cells expressing FLAG-SIRT6 were treated with 10 μm Bt-NO_2_-OA for 1 h. After cell lysis, Bt-NO_2_-OA adducts were affinity-purified using streptavidin-agarose beads followed by FLAG immunodetection (*lanes 1–4*). FLAG-SIRT6 from the same input lysates used for affinity purification is shown on the *right* (*lanes 5-8*). Immunoprecipitation was performed in HEK293T cells overexpressing FLAG-SIRT6 (*lanes 3* and *4*) and in control HEK293T cells without transfection (*lanes 1* and *2*). *Arrows* indicate 55- and 36-kDa molecular mass standards. *B,* HEK293T cells were treated with 10 μm OA or 10 μm NO_2_-OA for 1 h in serum-free DMEM and H3K9 acetylation assessed by Western blotting analysis. *C,* densitometric H3K9Ac quantification *versus* total amount of H3 (H3K9Ac/H3). Statistically significant differences were observed between control and NO_2_-OA incubation. *Error bars* represent S.D. of at least three replicates. **, *p* < 0.001 as determined by ANOVA.

To determine whether the reported interaction between NO_2_-OA and hSIRT6 could take place in a cellular context, HEK293T cells overexpressing FLAG-hSIRT6 were treated with 10 μm Bt-NO_2_-OA for 1 h. At the end of the incubation period, Bt-NO_2_-OA-modified proteins were pulled down from whole-cell lysates using streptavidin-conjugated beads and subjected to Western blotting analysis. As show in Fig. 9*A*, pulldown of Bt-NO_2_-OA resulted in prominent FLAG-SIRT6 detection, confirming the occurrence of this modification under cellular conditions.

### Nitro-fatty acids induced cellular histone deacetylation

The modulation of the cellular deacetylase activity of hSIRT6 was monitored by assessing changes in H3K9 acetylation levels following 1 h treatment of HEK293T cells overexpressing FLAG-hSIRT6 with OA or NO_2_-OA (10 μm). In accordance with the potent activation of hSIRT6 deacetylase activity observed *in vitro*, Western blotting analysis showed a marked decrease in H3K9Ac levels in HEK293T cells incubated with NO_2_-OA (Fig. 9, *B* and *C*). No decrease in H3K9Ac levels was observed upon OA incubation, with H3K9Ac/H3 ratios being identical to control levels. Taken together, these results show that NO_2_-OA effectively interacts with hSIRT6 in the cellular environment resulting in the activation of its deacetylase activity and the corresponding decrease in H3K9 acetylation levels.

## Discussion

The hydrophobic pocket in the active site of hSIRT6 accommodates a long acyl-chain resulting in better deacylase than deacetylase activity *in vitro*. Previous studies observed that free fatty acids bound to this hydrophobic crevice cause a conformational change that brings closer the two domains of the enzyme, which in turn favors the binding of the substrates. Thus, the poor *in vitro* deacetylase activity of SIRT6 increases in the presence of free fatty acids such as OA. Considering the enzyme depends on a histidine residue (His-133) for catalysis, we speculated that electrophilic NO_2_-FA would accommodate in the active site, react with the nucleophilic histidine, and inhibit SIRT6 activity. Surprisingly, incubation with low micromolar concentrations of NO_2_-OA or NO_2_-CLA not only resulted in activation of the deacetylase activity but also to a significantly higher degree than that obtained with the corresponding non-nitrated fatty acids (Fig. 2). Molecular docking analyses support the experimental results showing that once accommodated in the active site, the distance and orientation of the reactive nitroalkene moiety do not allow for reaction with the catalytic histidine (Fig. 7). Nevertheless, UV-visible determinations showed that NO_2_-CLA was consumed upon incubation with hSIRT6 suggesting that a reaction with nucleophilic residues of the enzyme was taking place (Fig. 4). MS analysis confirmed Michael adduct formation with cysteine residues, in particular with Cys-141 (coordinating Zn^2+^) and Cys-18 at the N terminus (Fig. 5). Interaction of SIRT6 with NO_2_-OA at the cellular level was confirmed by pulldown with streptavidin after incubating HEK293T cells overexpressing FLAG-SIRT6 with Bt-NO_2_-OA (Fig. 9). Despite not having a catalytic cysteine, oxidation of sirtuin cysteines has been shown to affect hSIRT6 activity *in vitro* and *in vivo* (62). For example, Cys-18 was found to be *S*-sulfenylated when colon cancer cells were exposed to hydrogen peroxide, resulting in the formation of a covalent complex with HIF1α via disulfide bonding (63). Similarly, work on lipopolysaccharide-activated human monocytes demonstrated SIRT6 sulfenylation on Cys-144 (64), and incubation of hSIRT6 with nitrosating agents resulted in protein *S*-nitrosation and loss of demyristoylase activity (65).

Molecular docking analysis (Fig. 7) showed that a NO_2_-FA molecule accommodated in the active site would be unable to react with any of these cysteine residues, indicating that Michael adduct formation likely involves a different reaction site. Importantly, the covalent modification of hSIRT6 by NO_2_-FA induces a conformational change (Fig. 3) that results in a much larger increase in enzymatic activity than that attained by mere binding of the long acyl chain of the NO_2_-FA on the active site loop (Fig. 2). In line with this concept, incubation of NO_2_-FA with the mutant C18S resulted in only a minor activation (“the fatty acid effect”) compared with the much stronger effect achieved with the *wt* hSIRT6 where the Michael adduct with Cys-18 can be formed (Fig. 6). This result also indicates that, although Cys-141 is susceptible to modification by NO_2_-FA, this reaction is not responsible for hSIRT6 activation. On the whole, the results suggest that NO_2_-FA may still activate hSIRT6 by binding to the active site in a similar fashion as non-nitrated fatty acids, the formation of a Michael adduct with Cys-18 induces a much greater activation as a result of the stabilization of the hydrophobic patch constituted by residues Met-3, Tyr-5, Pro-11, Tyr-12, Ala-13, and Lys-15 in the acyl/co-substrate–binding loop (Fig. 8). Both activation mechanisms are expected to be independent and thus could not be mediated by the same NO_2_-FA molecule. SIRT6 has the shortest N-terminal region among the seven sirtuins and is the only isoform that contains a reactive cysteine in this region (30), suggesting that the potent activation induced by NO_2_-FA is likely to be specific to SIRT6. In fact, no *in vitro* activation of SIRT1 was observed by NO_2_-FA.

Treatment of HEK293T cells with NO_2_-OA resulted in a significant decrease in the levels of acetylated H3K9, an effect that was not observed when equivalent concentrations of OA were used (Fig. 9, *B* and *C*). Importantly, the concentration of NO_2_-OA required to activate cellular hSIRT6 (10 μm) is comparable with those shown to elicit physiologically relevant signaling events *in vitro* that then translate successfully to both animal and human studies (52, 66, 67). In this regard, administration of NO_2_-OA to both genetic and dietary mouse models of obesity results in improved insulin sensitivity and glucose tolerance, as well as in protection against mitochondrial dysfunction (68, 69). These effects have been ascribed to a combination of partial peroxisome proliferator-activated receptor γ agonism, inhibition of pro-inflammatory NF-κB signaling, attenuation of reactive species generation, and prevention of mitochondrial dysfunction in the adipose tissue (68, 69, 70, 71). Notably, these results are also consistent with those obtained upon SIRT6 overexpression in both obese and non-obese mouse models (13, 14, 15), suggesting that posttranslational SIRT6 activation could contribute to the metabolic effects of NO_2_-OA *in vivo*.

Considering that SIRT6 is a key enzyme in inflammation and metabolism, effective modulators of its activity are of special interest. Herein we report that nitrated derivatives from known fatty acid activators of hSIRT6 are able to further increase enzyme activity by ∼40-fold. We postulate that a synergistic mechanism may be at play, involving both the binding of a NO_2_-FA to a pocket in the active site of the enzyme and the formation of a Michael adduct with Cys-18. The complementary effect of Cys-18 nitro-alkylation may shift the N-terminal region of hSIRT6 to its active conformation, similar to other activators, such as MDL-800/801 (45), UBSC039 (44), quercetin, isoquercetin, and cyanidin (48). Taken together, our results indicate that activation of SIRT6-dependent deacetylase activity could significantly contribute to the physiological and pharmacological actions of NO_2_-FA *in vivo*.

## Experimental procedures

### Reagents

Acylpeptide H3K9 (Ac-KQTARKSTGGWW), H3K9Ac (Ac-KQTARK(Ac)STGGWW), and H3K9Myr (Ac-KQTARK(Myr)STGGWW) were custom synthesized at ≥90% purity by United Biosystems, VA, USA. 9- and 10-Nitrooctadec-9-enoic acid (nitro-oleic acid, 9- and 10-NO_2_-OA), 9- and 12-nitrooctadec-9,11-dienoic acid (nitro-conjugated linoleic acid, 9- and 12-NO_2_-CLA), and Bt-NO_2_-OA were synthesized as previously described (58, 72, 73). For both, NO_2_-OA and NO_2_-CLA, a 1:1 mixture of the positional isomers in methanol (2 mm) was used as stock and kept at −80 °C. Cell media, isopropyl β-d-1-thiogalactopyranoside (IPTG), β-mercaptoethanol (β-ME), and tris(2-carboxyethyl)phosphine hydrochloride (TCEP) were purchased from Sigma-Aldrich, USA. All other chemicals were of analytical grade.

### Recombinant hSIRT6 WT and hSIRT6 C18S expression and purification

Competent BL21-DE3 *Escherichia coli* cells were transformed with pQE-80L plasmid containing N-terminal His-tagged human SIRT6 sequence (1-355) or His-tagged human SIRT6 C18S and were grown in 2YT medium supplemented with 100 μg/ml of ampicillin to an absorbance of 0.6 at 600 nm. Protein expression was induced overnight by addition of 0.5 mm IPTG at 25 °C. Cells were harvested by centrifugation at 5000 × *g* and frozen at −80 °C. Frozen cell pellets were thawed on ice and resuspended in lysis buffer: 50 mm sodium phosphate, pH 7.2, containing 250 mm NaCl, 5 mm imidazole, and 1 mm β-ME. Cells were lysed via sonication, centrifuged (18,000 × *g*), and applied to a nickel-nitrilotriacetic acid column (GE Healthcare), washed with 5 volumes of lysis buffer followed by 5 volumes of 50 mm sodium phosphate, pH 7.5, containing 250 mm NaCl, 10 mm imidazole, and 1 mm β-ME. Elution was carried out using 250 mm imidazole in the buffer. hSIRT6 was further purified by cationic exchange on a HiTrap SP-Sepharose Fast Flow column (GE Healthcare) in 50 mm sodium phosphate, pH 7.2, 50 mm NaCl, and 1 mm β-ME. After washing the column with 150 mm NaCl in buffer, elution was carried out by a linear gradient from 150 to 750 mm NaCl in the same buffer. Fractions containing purified hSIRT6 (containing 1 mol of Zn^2+^/mol of protein) were concentrated and dialyzed into 50 mm Tris, pH 8.0 (4 °C), 150 mm NaCl, 100 μm TCEP, and 5% (w/v) glycerol, and stored at −80 °C.

### Recombinant Pnc1 expression and purification

Competent BL21-DE3 *E. coli* cells were transformed with pET28a plasmid containing His-tagged Pnc1 nicotinamidase from *Saccharomyces cerevisia*e (74) and grown in LB medium supplemented with 35 μg/ml of kanamycin to an absorbance of 0.6 at 600 nm. Protein expression was induced by addition of 1 mm IPTG for 4 h at 37 °C. Cells were harvested (5000 × *g*) and frozen at −80 °C. Frozen cell pellets were thawed on ice and resuspended in lysis buffer: 50 mm Tris, pH 7.2, 250 mm NaCl, 5 mm imidazole, and 1 mm β-ME. Recombinant protein was purified from the clarified cell lysate by nickel affinity chromatography. After loading the column, a 10-volume wash was carried out with 50 mm Tris, pH 7.2, 250 mm NaCl, 30 mm imidazole, and 1 mm β-ME. For the elution, 500 mm imidazole was used in the same buffer. Pnc1 was further purified by gel filtration in 50 mm sodium phosphate, pH 8, 150 mm NaCl, 3 mm β-ME and stored at −80 °C.

### Quantification of Zn^2+^

To determine the stoichiometry of zinc bound to hSIRT6, the metal ion was first released by *p*-chloromercurybenzoate treatment and complexed with the dye 4-(2-pyridylazo)resorcinol (PAR). A molar extinction coefficient of 66,000 m^−1^ cm^−1^ at 500 nm was used for the resulting Zn-PAR complex (75). The reaction mixture contained 2 μm hSIRT6, 20 μm *p*-chloromercurybenzoate, 40 μm PAR in 50 mm sodium phosphate, pH 7.4, and absorbance at 500 nm was monitored.

### Deacetylase activity assay

hSIRT6 deacetylase activity was measured under initial rate conditions using an enzyme-coupled assay as previously reported (76). Assays were performed in 50 mm sodium phosphate, pH 7.4, 150 mm NaCl, at 37 °C, in a final volume of 100 μl. The reaction mixture contained 0.6 mm NAD, 3 mm α-ketogutarate, 0.2 mm NADPH, 3 μm Pnc1, 2 units of glutamic dehydrogenase, 50 μm H3K9Ac, and 0.5 μm hSIRT6 or hSIRT6 C18S. The reaction mixture was preincubated for 20 min at 37 °C until absorbance at 340 nm was stable and deacetylation reaction started by the addition of hSIRT6 or hSIRT6 C18S.

### Effect of nitrated and non-nitrated fatty acids on hSIRT6 deacetylase activity

To determine the effect of fatty acids or nitro-fatty acids on deacetylase hSIRT6 activity, the enzyme (WT or mutant, 1 μm) was preincubated, for 30 min at room temperature, with variable excess concentrations of OA, CLA, and their respective nitro-derivatives (NO_2_-OA, NO_2_-CLA). Racemic mixtures of the nitro-fatty acids used were: 9- and 10-NO_2_-OA, and 9- and 12-NO_2_-CLA (see Fig. 2). Stock solutions of the fatty acids in methanol were diluted in DMSO immediately before the experiment. All fatty acid concentrations used were below the CMC (CMC for OA, 150-200 μm (43), CMC for NO_2_-OA 40-60 μm).^6^ Nitro-fatty acid concentrations were determined using ε_270 nm_ = 8220 m^−1^ cm^−1^ and ε_330nm_ = 6,490 m^−1^ cm^−1^ (in phosphate buffer, pH 7.4) for NO_2_-OA and NO_2_-CLA, respectively (77). Excess fatty acid was eliminated by gel filtration before activity measured using the enzyme- coupled assay.

### Structural assessment by CD

Protein samples in 10 mm phosphate buffer, pH 7.4, were filtered (0.2 μm) before running the spectra on a Chirascan Q100 spectropolarimeter (Applied Photophysics, UK). A 3 μm protein concentration was used for far-UV CD (0.1-cm light path) and 10 μm for near-UV CD (1-cm light path cuvette). All spectra were normalized using: ellipticity ϴ (mdeg)/(*n*-1) × (μm protein) × 10 light path (cm), where *n* is the number of amino acid residues. hSIRT6 was incubated at 37 °C for 30 min in the absence or presence of 5-fold excess of OA or NO_2_-OA before running the spectra.

### In vitro hSIRT6 modification by biotinylated NO_2_-OA

hSIRT6 was first reduced in batch using a TCEP resin (30 min at 25 °C) followed by incubation with 10 mm NEM or 1 mm DEPC for 30 min at 37 °C to block cysteines and histidines, respectively. Unreacted reagents were removed by gel filtration and protein concentration was determined by UV absorbance (ε_280_ = 33,460 m^−1^ cm^−1^). Variable concentrations of Bt-NO_2_-OA were incubated with 1.2 μm hSIRT6 for 30 min at 25 °C, followed by SDS-PAGE separation and transfer to a PVDF membrane (1 h, 4 °C). Loaded amounts of hSIRT6 were visualized with Ponceau S staining. Membrane was blocked with PBS, 1% casein, overnight at 4 °C, and incubated with streptavidin-HRP for 1 h at room temperature. After three washes in 0.1% TBS-T buffer, bands were visualized using an ECL Western blotting system (ThermoFisher Scientific). Band intensities were quantified using ImageJ.

### ESI-MS/MS analysis of Michael adduct formation between hSIRT6 and NO_2_-OA

hSIRT6 was reduced with 5 mm DTT (30 min at room temperature) and excess DTT was removed by gel filtration. Reduced hSIRT6 (4 μm) in 100 mm ammonium bicarbonate, pH 8, was incubated with two concentrations of NO_2_-OA (0.5 and 5×) for 30 min at 25 °C, and unreacted NO_2_-OA was removed by gel filtration to avoid interference during the analysis. Tryptic digestion was carried out for 2 h at 37 °C and the resulting peptides were analyzed by nano-LC (EASY-nLC 1000, Thermo Scientific) coupled to a linear ion trap mass spectrometer (LTQ Velos, Thermo Scientific). Peptide separation was performed using an in-house packed reverse phase nanocolumn (ReproSil-Pur®, Maisch; inner diameter, 75 mm; length, 28 cm) at a flow rate of 300 nl/min. A 70-min gradient of 0.1% (v/v) formic acid in water, 0.1% (v/v) formic acid in acetonitrile was used (4–55% acetonitrile in 60 min; 55–100% in 10 min). Mass spectrometer was set in a data-dependent acquisition mode (full MS followed by MS/MS of 10 most intense ions, using a 30-s dynamic exclusion list). Peptides were identified by searching a database containing *E. coli* Uniprot protein sequences (downloaded September 2018) plus hSirt6 sequence. Proteome Discoverer software (version 1.3.0.339, Thermo) with Sequest as search engine were used with the following parameters: precursor mass tolerance: 1.5 Da; fragment mass tolerance 0.8 Da; oxidation of methionine and modification with NO_2_-OA (+327.24 Da) as dynamic modifications. High-confident peptides (target FDR set to 0.01) were considered. Manual inspection of the MS/MS spectra was performed to corroborate peptide sequence and modification site assignments.

### Intracellular SIRT6 modification by NO_2_-OA

HEK293T cells were grown in DMEM (high-glucose), 10% FBS, 100 units/ml of penicillin, and 100 μg/ml of streptomycin. Cells were transfected with FLAG-SIRT6 plasmid (Addgene plasmid number 13817) with Lipofectamine 2000 (Thermo Fisher Scientific) for 48 h. Cells were then incubated with 10 μm Bt-NO_2_-OA in serum-free DMEM for 1 h. Cell lysis and immunoprecipitation was performed as described previously (78). Briefly, cells were lysed in NETN buffer (20 mm Tris-HCl, pH 8.0, 100 mm NaCl, 1 mm EDTA, 0.5% Nonidet P-40) supplemented with 5 mm NaF, 50 mm 2-glycerophosphate, and a protease inhibitor mixture (Roche). Homogenates were incubated at 4 °C for 20 min under constant agitation and then centrifuged at 10,000 × *g* for 10 min at 4 °C. For each immunoprecipitation, 1 mg of protein was used. Samples were incubated with 20 μl of Strep-Tactin Sepharose beads for 1–2 h at 4 °C under constant rotation. Finally, immunoprecipitates were washed 2–3 times with cold NETN before addition of 2× Laemmli buffer followed by SDS-PAGE separation and transfer to a PVDF membrane (1 h, 4 °C). Primary and secondary antibodies utilized were anti-FLAG (Sigma, number F3165) and HRP-conjugated IgG from mouse (Sigma, A9044), respectively. Bands were visualized using an ECL Western blotting system (ThermoFisher Scientific) and images were processed by Photoshop.

### Assessment of cellular H3K9 acetylation

HEK293T cells were grown in DMEM (high-glucose), 10% FBS, 100 units/ml of penicillin, and 100 μg/ml of streptomycin. Cells were transfected with FLAG-SIRT6 plasmid (Addgene plasmid number 13817) with Lipofectamine 2000 (Thermo Fisher Scientific) for 48 h. Cells were later incubated with 10 μm NO_2_-OA (or 10 μm OA) in serum-free DMEM for 1 h. Cell lysis was performed as described previously (79). Briefly, cells were lysed in RIPA buffer (25 mm Tris, pH 8.0, 150 mm NaCl, 1% Nonidet P-40, 0.1% SDS) supplemented with 5 mm NaF, 50 mm 2-glycerophosphate, 1 μm trichostatin A, 5 mm nicotinamide, and a protease inhibitor mixture (Roche). Homogenates were incubated at 4 °C for 20 min under constant agitation and then centrifuged at 10,000 × *g* for 10 min at 4 °C. Protein lysates were separated on 15% SDS-PAGE followed by transfer to PVDF membrane and incubation with specific primary antibodies (H3K9Ac, H3, FLAG, and tubulin). H3K9Ac and H3 antibodies were from Cell Signaling (9649S and 4620S, respectively). Images were processed using Photoshop and densitometry analysis were done with ImageJ (Rasband, W.S., Bethesda, MD, USA).

### Computational modeling

The experimental holo structure of hSIRT6 in complex with a H3K9 myristoyl peptide and ADP-ribose fragment of NAD^+^ co-substrate (PDB code. 3ZG6 (28)) was used as receptor template. Residues 1 to 12 constituting the acyl-binding loop of hSIRT6 were removed from the structure due to their high flexibility, which is evidenced in apo structures (PDB codes 3PKI (34) and 5X16 (47)) or in presence of inhibitor/activator compounds (46, 80, 81). The co-substrate and water molecules 2006 2018 2019 2035 2036 2038 2040 2042 2053 2080 2099 2103 2104 2110 2111 2123 2124 2132 were kept in the structure to mask unspecific pockets around the protein surface. The coordinates of the receptor were moved prior docking according to the following transformation matrix using VMD 1.9.3 (82): {-0.782000720500946 0.30951544642448425-0.5409945249557495 69.48826599121094} {0.5513688921928406-0.06120478734374046-0.8320134282112122 3.671846389770508} {-0.2906324565410614-0.9489226341247559-0.1227950006723404-6.218472480773926} {0.0 0.0 0.0 1.0}, where braces define row vectors. The ligands corresponded to OA, 9-NO_2_-OA, 10-NO_2_-OA, CLA, 9-NO_2_-CLA, and 12-NO_2_-CLA (Fig. 2*A*). Docking calculations were done with AutoDock Vina (83). Each molecule was prepared with AutoDockTools (84) by merging nonpolar hydrogen atoms and computing Gasteiger charges. Histidine residues were assumed to be protonated at Nε. The searching grid was centered at coordinates *x* = 21.464 Å, *y* = 3.555 Å, and *z* = 16.125 Å, and the total size was defined as 50, 70, and 44 Å for each coordinate, respectively. The receptor was considered rigid while ligands were allowed to be full flexible. At each run 20 binding modes were generates using an exhaustiveness of 20 and allowing for energy ranges of 100 kcal/mol. Calculations were replicated 500 times for a total of 10,000 poses per ligand. Poses scoring up to 1 kcal/mol from the best conformation were selected for cluster analysis. Cluster analysis was based on the distance matrix of root mean square deviations, using a cut-off of 4 Å for grouping poses. On each case, the best pose from the top populated cluster was chosen for analysis. Possible conformations for 9-NO_2_-OA conjugation with Cys-18 were manually build and sculpted using PyMOL 1.8.4.0 (RRID:SCR_000305).

## Statistical analysis

Data are presented as mean ± S.D. (experiments repeated at least three times). Statistical comparisons were made using one way-ANOVA followed by Tukey's multiple comparison test (GraphPad Prism 8.02). Difference was considered significant if *p* < 0.05.

## Data availability

All data are contained within the manuscript. Raw data are available upon request (denicola@fcien.edu.uy, escande@pasteur.edu.uy).

## Footnotes

6 M. Carreño and A. Denicola, unpublished results.
